# Machine learning approaches linking brain function to behavior in the ABCD STOP task

**DOI:** 10.1002/hbm.26172

**Published:** 2022-12-19

**Authors:** Dekang Yuan, Sage Hahn, Nicholas Allgaier, Max M. Owens, Bader Chaarani, Alexandra Potter, Hugh Garavan

**Affiliations:** ^1^ Department of Psychiatry University of Vermont Burlington Vermont USA

**Keywords:** adolescence, big data, fMRI, important feature, machine learning, multimodality, stop‐signal reaction time, stop‐signal task

## Abstract

The stop‐signal task (SST) is one of the most common fMRI tasks of response inhibition, and its performance measure, the stop‐signal reaction‐time (SSRT), is broadly used as a measure of cognitive control processes. The neurobiology underlying individual or clinical differences in response inhibition remain unclear, consistent with the general pattern of quite modest brain–behavior associations that have been recently reported in well‐powered large‐sample studies. Here, we investigated the potential of multivariate, machine learning (ML) methods to improve the estimation of individual differences in SSRT with multimodal structural and functional region of interest‐level neuroimaging data from 9‐ to 11‐year‐olds children in the ABCD Study. Six ML algorithms were assessed across modalities and fMRI tasks. We verified that SST activation performed best in predicting SSRT among multiple modalities including morphological MRI (cortical surface area/thickness), diffusion tensor imaging, and fMRI task activations, and then showed that SST activation explained 12% of the variance in SSRT using cross‐validation and out‐of‐sample lockbox data sets (*n* = 7298). Brain regions that were more active during the task and that showed more interindividual variation in activation were better at capturing individual differences in performance on the task, but this was only true for activations when successfully inhibiting. Cortical regions outperformed subcortical areas in explaining individual differences but the two hemispheres performed equally well. These results demonstrate that the detection of reproducible links between brain function and performance can be improved with multivariate approaches and give insight into a number of brain systems contributing to individual differences in this fundamental cognitive control process.

## Graphical abstract

We investigated the potential of multivariate, machine learning (ML) methods to improve the estimation of individual differences in stop‐signal reaction time (SSRT) with region of interest‐level multimodal structural and functional neuroimaging data from the ABCD Study. Six ML algorithms were assessed across modalities and fMRI tasks. As expected, SSRT was best explained by stop‐signal task activation with 12% of individual differences in SSRT explained in cross‐validation and out‐of‐sample lockbox data sets. Brain regions that were more active during the task and that showed more interindividual variation in activation were better at capturing individual differences in performance on the task, but this was only true for activations when successfully inhibiting.

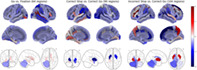

## 1 INTRODUCTION

Response inhibition is a crucial cognitive ability for a changing and complex environment (Braet et al., 2011). Its functional neuroanatomy has been investigated in many previous studies (Aron & Poldrack, 2006; Boehler et al., 2010; Cai & Leung, 2011; Garavan et al., 1999; Hampshire et al., 2010; Konishi et al., 1999; Sharp et al., 2010), which have identified several regional brain correlates such as the right inferior frontal gyrus (IFG), pre‐supplementary motor area (SMA), dorsal anterior cingulate cortex (dACC), and basal ganglia (Cai & Leung, 2011; Rae et al., 2014; Rae et al., 2015). Researchers have shown using transcranial magnetic stimulation that the right IFG is critical to performance on the stop‐signal task (SST), which is a cognitive control task requiring the inhibition of prepotent motor responses (Chambers et al., 2006). In agreement with such findings, fMRI studies have also detected activations in the posterior IFG during the SST (Aron et al., 2007; Cai & Leung, 2009; Cai & Leung, 2011; Congdon et al., 2010; Coxon et al., 2009; Leung & Cai, 2007; Rubia et al., 2001; Rubia et al., 2003). Performance on the SST can be assessed using the stop‐signal reaction‐time (SSRT), which measures the duration of time required to inhibit a “go” response after a “stop signal” has been presented. SSRT for the present study was computed using the integration method (Verbruggen & Logan, 2009) with assumptions of the race model from Logan and Cowan's (1984) study. It indicates that SSRT is computed by subtracting the average stop‐signal delay (SSD) from the finishing time of the stop process which is determined by the go response time distribution (specifically, the *n*th go response time in which *n* is the proportion of stop trials on which the participant successfully inhibited).

SSRT has been broadly applied as a clinically relevant measurement of inhibitory control, most often in studies of attention deficit and hyperactivity disorder which have been associated with slower SSRTs (Eagle et al., 2008; Logan et al., 1997; Oosterlaan et al., 1998; Rubia et al., 1998). Other investigations have linked slower SSRTs to disorders such as obsessive–compulsive disorder (Eagle et al., 2008; Krikorian et al., 2004), Parkinson's disease (Eagle et al., 2008; Gauggel et al., 2004), schizophrenia (Hughes et al., 2012), substance use disorders (Alcorn III et al., 2017; Li et al., 2006; Weafer et al., 2014), treatment response (Maguire et al., 2018), as well as adolescent impulsivity (Whelan et al., 2012). Given the SST's prominence as a standard and common assay for inhibitory control abilities, it may provide insight into the neurobiology that underlies interindividual variation in inhibitory control. Understanding the neurobiology underlying individual differences in SSRT would yield insight into variation across people in their cognitive control abilities which could, in turn, inform diagnosis and treatment.

A previous well‐powered analysis of the SST has shown widespread brain activations correlated with SSRT. A mass univariate approach reported that greater activation in insula and the dlPFC correlated with faster SSRT, but the single vertex (lateral thalamic voxels) with the strongest association explained no more than 2% of the variance in SSRT (Chaarani et al., 2021). In this prior publication, Chaarani et al. calculated the absolute maximum Pearson's correlation coefficient for both cortical and subcortical activation measures with a mass‐univariate approach and with no cross‐validation. Indeed, there is a growing appreciation that small brain–behavior association effect sizes are to be expected in neuroimaging research and that many extant findings reflect spuriously large effects based on unreliably small samples (Marek et al., 2020). Moreover, in Marek et al.'s (2020) study, the replicability of univariate analysis is generally less than multivariate analysis with large sample size. Working with the large and well‐characterized ABCD data set, Owens et al. showed the median behavior–behavior association to be *r* = .03 (Owens et al., 2021) in a correlation analysis among 161 variables. Further, Marek et al. report a median absolute effect size of *r* = .01 when relating cortical thickness or resting‐state edge measures to composite cognitive and mental health measures (Marek et al., 2020). The magnitudes of these effects underscore the improved performance obtained by applying the multivariate approaches investigated in the present study. The present results hold promise that multivariate approaches, perhaps combined with other methods to improve brain activation sensitivity and reliability, will yield improved insights into the neurobiological correlates (Stern et al., 2005) of individual differences in cognitive abilities (Sripada et al., 2020).

With the growing availability of large‐sample neuroimaging data sets, there exists the opportunity to apply machine learning (ML) models to discover complex and subtle relationships between the brain and behavior. By segregating a large sample into independent model development and model testing subsets, ML methods can identify potential novel associations in an exploratory fashion and then, importantly, assess if these associations are generalizable. Regression‐based methods have been a popular approach for multivariate analyses, such as linear, ridge, least absolute shrinkage and selection operator (LASSO), elastic net, support vector machine (SVM; Cui & Gong, 2018; Salvador et al., 2017), random forest (Kesler et al., 2017), and gradient boosting (Salvador et al., 2019), some of which are capable of incorporating nonlinear relationships, and all of which can achieve similar or better levels of accuracy than standard linear regression models. Although ML modeling has already been remarkably productive in neuroimaging studies, limited sample sizes and imperfect model validation methodologies have hindered the performance of predictive analytics (Button et al., 2013; Vabalas et al., 2019). In addition, which ML algorithm might be most effective for neuroimaging studies is unclear: Jollans et al. (2019) demonstrated the impact that sample and feature set sizes have in head‐to‐head comparisons of different algorithms applied to neuroimaging data. In the present study, multiple cross‐validated ML models predicting SSRT were built employing the large sample and the rich neuroimaging characterization of the Adolescent Brain Cognitive Development Study (ABCD Study) (Casey et al., 2018).

Our objective was to determine how much out of sample variance in SSRT could be explained through use of multivariate ML methods applied to multimodal neuroimaging data. While our primary focus was on the SST we also explored whether the other probes of brain function and the measures of brain structure that are available in the ABCD test battery might contribute to explaining interindividual differences in SSRT. Furthermore, we aimed to characterize the relative importance of brain activation associated with the different psychological processes engaged during the SST (e.g., motor processes associated with go responses, motor inhibition associated with successful stop trials, monitoring processes associated with stop fail trials) in predicting SSRT and to identify important brain regions in predicting SSRT. In this study, we (1) tested whether SST brain activation would best estimate SSRT, compared with other imaging modalities and across different ML algorithms, (2) evaluated the relative predictive power of left and right hemisphere activation, cortical and subcortical activation, and the different activation contrasts derived from the SST, and (3) identified the most important features for explaining SSRT for each of the multivariate models.

## 2 MATERIALS AND METHODOLOGY

### 2.1 Participants

Predictive models were generated with data from the ABCD study, an ongoing longitudinal project that includes 11,875 youths recruited for their baseline assessment at ages 9 and 10 years (see sample demographics in Table 1).

**TABLE 1:** Mean and standard deviation of age, and the count and proportion of gender and race for all ABCD participants, participants included in the SST analyses, and analyses restricted to participants with data across all neuroimaging modalities

	ABCD all participants (*n* = 11,875)		SST only (*n* = 7296)		All‐modality participants (*n* = 3808)	
	Count	%	Count	%	Count	%
Child sex (male)	6188	52.14%	3643	49.97%	1877	50.62%
Child sex (female)	5681	47.86%	3653	50.07%	1831	49.37%
Race (White)	6174	52.08%	4125	56.58%	2275	61.35%
Race (Black)	1779	15.01%	840	11.52%	303	8.17%
Race (Asian)	252	2.12%	160	2.19%	87	2.35%
Race (Other)	1245	10.49%	741	10.16%	359	9.68%
Ethnicity (Hispanic)	2407	20.30%	1424	19.53%	684	18.45%
	Mean	SD	Mean	SD	Mean	SD
Age (months)	118.94	7.46	119.27	7.53	119.83	7.50

Abbreviations: ABCD, Adolescent Brain Cognitive Development; SST, stop‐signal task.

### 2.2 Data collection

We assessed how well SSRT could be predicted utilizing data from the full ABCD imaging battery, which includes: morphological MRI, diffusion tensor imaging (DTI), and fMRI for resting state, the stop‐signal task (SST), Monetary Incentive Delay (MID) task (assessing reward and loss anticipation and receipt), and an EN‐Back working memory task that contained place stimuli and face stimuli evincing different emotions and contained both 0‐Back and 2‐Back conditions (see Maguire et al., 2018 for task details). The SSRT represents the duration of time required to inhibit an action after a stop signal is shown during the SST. It was calculated for each participant as the difference between the mean SSD (the interval between the onset of the Go stimulus and Stop signal) and that participant's *n*th rank‐ordered Go reaction time, where *n* is the number of Go reaction times multiplied by the participant's overall failure rate (probability of responding) on stop trials (Whelan et al., 2012; White et al., 2014).

The brain measure features were extracted from each of the 148 cortical regions of interest (ROIs) in the Destrieux atlas (Destrieux et al., 2010), as well as 30 regions at the subcortical level including 8 bilateral structures (accumbens, amygdala, caudate, hippocampus, pallidum, putamen, thalamus, cerebellum) and other measures. The brain features included cortical thickness and surface area for each cortical ROI and gray matter volume for the subcortical ROI's. For DTI, average fractional anisotropy and mean diffusivity within the subadjacent white matter associated with each ROI on the Destrieux atlas was captured. For resting‐state fMRI, functional connectivity was captured within and between networks for each network in the Gordon parcellation (Gordon et al., 2016). The resting‐state data were sampled onto the cortical surfaces and divided into the 422 cortical parcels that assemble 13 functional networks described in Gordon et al. (2016) and Owens et al. (2020). Correlations were computed for the average timeseries of vertices in each ROI pair, and the average was taken of the z‐transformed correlations of all ROI pairs within each network to determine the intranetwork functional connectivity (Owens et al., 2020). The average z‐transformed correlations of all ROI pairs between two networks determined the between‐network functional connectivity.

For task fMRI, nine different contrasts were selected across the different tasks: from the SST, correct stop versus correct go (stop success contrast), incorrect stop versus correct go (stop fail contrast), and correct go versus fixation (go success contrast); from the MID, large loss versus neutral anticipation, large reward versus neutral anticipation, loss positive versus negative feedback, and reward positive versus negative feedback; from the EN‐Back, 2‐back versus fixation and 2‐Back versus 0‐Back. Although SST brain activations were anticipated to be most strongly related to SSRT, we sought to determine whether reward processing (MID) and working memory (EN‐Back) were also linked to inhibitory control. Previous work suggests that reward and punishment can have distinct effects on go and stop performance (Guitart‐Masip et al., 2012; Verbruggen et al., 2014) and working memory capacity has been shown to be associated with inhibitory control (Marton et al., 2007). Further, investigating the predictive value of tasks that engage many similar cortical and subcortical regions to the stop task (acknowledging that this overlap is at the resolution of fMRI activations) addresses the importance of the cognitive processes being engaged. That is, we anticipate that the specific tasks/processes that induce regional activation will be an essential element of the ability of brain activation to capture individual differences in inhibitory control.

Task‐related activation in each contrast was the average beta weights of all vertices within each cortical ROI and all voxels within subcortical ROIs. The beta weights were obtained from general linear modeling (GLM) of the time series of BOLD signals (Zhang & Li, 2012). GLM analysis and beta weight extraction into ROIs were conducted by the ABCD Data Analysis and Informatics Resource Center. For more information about MRI and fMRI preprocessing and first‐level GLM, see Hagler Jr et al. (2019).

### 2.3 Descriptive statistics of model features

**FIGURE 1:**
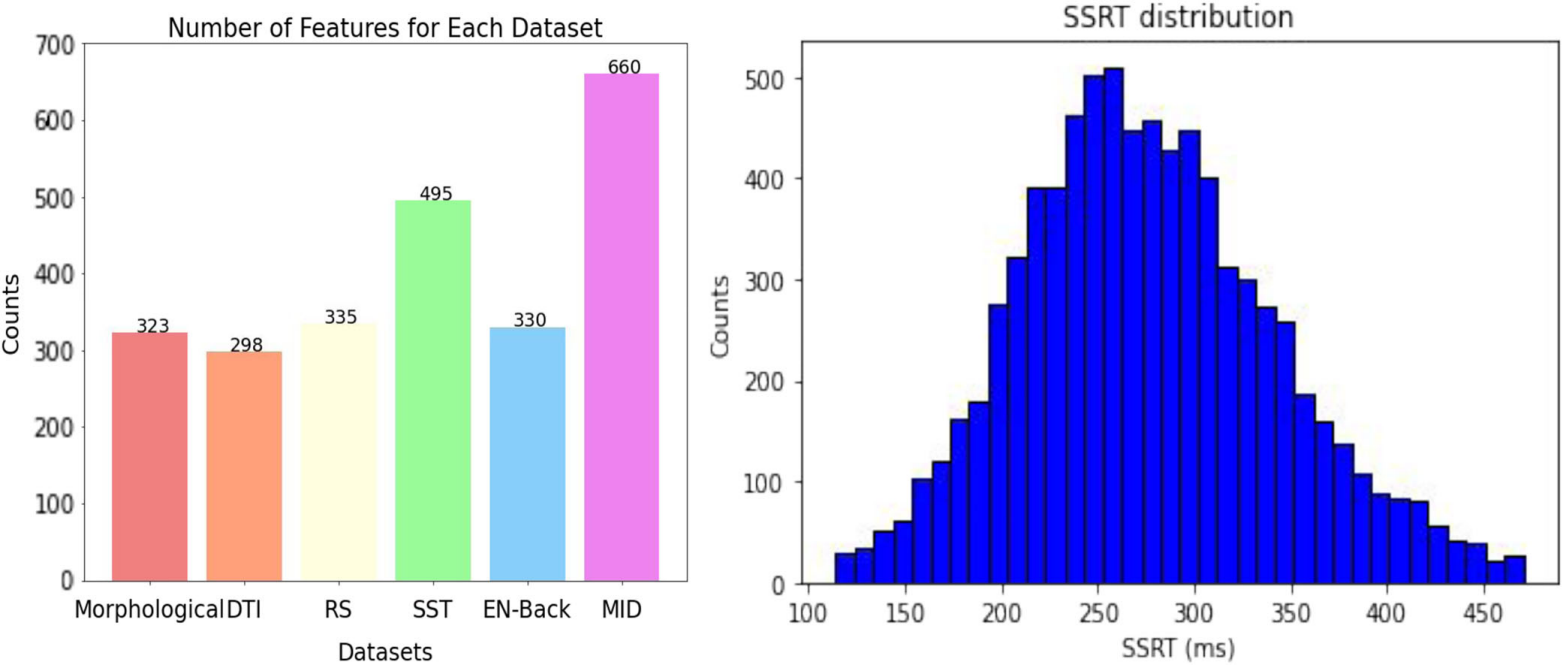
Number of features for each modality (left); and the distribution of stop‐signal reaction‐time (SSRT) (right)

Because the performance of ML algorithms is related to sample size, it is beneficial for a comparison among models to have the same number of participants. Therefore, in analyses comparing across MRI modalities, listwise deletion was applied to deal with missingness for all 2368 variables used as features, resulting in the inclusion of 3708 participants with data for all modalities (see Figure 1). However, for the internal comparisons in the SST fMRI data set, there were 9542 subjects who had full and valid data available for the three contrasts in the 165 ROIs. After data cleaning (see details on exclusions in Supplementary Table S1), the final number of participants for SST was 7298. Participants who violated the race model assumption by having a faster mean Go RT than mean stop fail RT were excluded (Verbruggen et al., 2019). Figure 1 shows the number of features in each MRI paradigm as well as the distribution of the SSRT.

### 2.4 Machine learning algorithms

All ML approaches were implemented in Python using Scikit‐Learn (Pedregosa et al., 2011) and the Brain Predictability toolbox (Hahn et al., 2020). In the following subsections, the ML algorithms are briefly described.

#### 2.4.1 Linear regression

Simple linear regression describes the relationship between a dependent variable and a collection of features using linear combinations of the latter. A simple linear regression model is based on an objective function with goal of minimizing the sum of squared errors, which is equivalent to:

$$\mathrm{Min}\;\sum_{i=1}^{n}{\left(y_{i}-\sum_{j=1}^{p}x_{\mathrm{ij}}\beta_{j}-\beta_{0}\right)}^{2}$$

In this equation, *x* represents the features as a matrix and β is the vector containing coefficients for each feature including $\beta_{0}$ as an intercept. The aim of such a regression model is to minimize the residual sum of squares between the observed and predicted outputs.

#### 2.4.2 Ridge regression

Standard linear regression performs poorly in a situation with multicollinearity. An alternative is regularized regression, wherein a constraint is placed on the model coefficients to be regressed. A few methods exist to shrink the magnitude of regression coefficients (Awad & Khanna, 2015), which can reduce associated overfitting. One approach is to constrain the sum of the squared regression coefficients to be less than some constant, and obtains regression coefficients by:

$$\hat{\beta }={\mathrm{Min}}_{\beta }\left[\sum_{i=1}^{n}{\left(y_{i}-\beta_{0}-\sum_{j=1}^{p}x_{\mathrm{ij}}\beta_{j}\right)}^{2}+\lambda \sum_{j=1}^{p}\beta_{j}^{2}\right]$$

where we have now introduced a penalty derived from the magnitude of the coefficient estimates: a larger coefficient would be subject to a heavier penalty. *λ* is a tuning hyperparameter that regulates the level of penalty. Ridge regression handles multicollinearity by assigning predictive (but correlated) features coefficients of similar magnitude.

#### 2.4.3 LASSO regression

The LASSO is another regularization technique, penalizing the sum of the absolute values of the regression coefficients to be less than some constant, $L_{1}$ penalty, and can be described as:

$$\hat{\beta }={\mathrm{Min}}_{\beta }\left[\sum_{i=1}^{n}{\left(y_{i}-\beta_{0}-\sum_{j=1}^{p}x_{\mathrm{ij}}\beta_{j}\right)}^{2}+\lambda \sum_{j=1}^{p}\left|\beta_{j}\right|\right]$$

Similar to ridge regression, a *λ* value of zero produces the basic ordinary least‐squares equation, however given a suitable *λ* value LASSO regression can drive some coefficients to zero. A larger *λ* would lead to more features being shrunk to zero. Such a process can eliminate some features by setting coefficients as zero and yields a subset of features that helps mitigate multicollinearity and model complexity. Predictors not shrunk toward zero signify that they are important and thus $L_{1}$ regularization allows for feature selection.

#### 2.4.4 Elastic net regression

Ridge regression is considered superior if there are many parameters of about the same value, that is, when many predictors truly impact the target. LASSO, on the other hand, performs well when there are a small number of significant parameters with the others being closer to zero, that is, when only a few predictors actually influence the target. Elastic net regression combines the penalties of ridge and LASSO regression. Elastic net has the following formula:

$$\hat{\beta }={\mathrm{Min}}_{\beta }\left[\sum_{i=1}^{n}{\left(y_{i}-\beta_{0}-\sum_{j=1}^{p}x_{\mathrm{ij}}\beta_{j}\right)}^{2}+\lambda_{1}\sum_{j=1}^{p}|\beta_{j}|+\lambda_{2}\sum_{j=1}^{p}\beta_{j}^{2}\right]$$

LASSO regression is capable of eliminating non‐informative features, and reducing overfitting in a predictive linear model. Ridge regression can reduce the impact of features that are not important in predicting dependent variables, and also groups predictive but correlated features. Elastic net regression combines the two in an effort to obtain the best of both worlds.

#### 2.4.5 SVM regression

SVM is a commonly applied classification technique using the concept of a maximum margin hyperplane in feature space, where the margin, *γ*, is the distance from the hyperplane (like a boundary) to the closest points in either class. However, SVM algorithms have been developed for a numeric prediction (i.e., regression) that share many of the properties encountered in the classification case: they produce a model that can usually be expressed in terms of a few support vectors and can be applied to nonlinear problems using kernel functions. It is commonly applied with different kernel functions to map the original input variables into high‐dimensional feature space which allows the incorporation of nonlinearity in the solution. Given a set of data points, an SVM regression model relates the output variable and the input variables as:

$$Y=f\left(X\right)=W\times \psi \left(X\right)+b$$

where $\psi \left(X\right)$ corresponds to the features mapped from the original input variable with a kernel function. SVM extension to SVR is realized by introducing an ε‐insensitive region around the function, which is referred to as the ε‐tube that best approximates the regression function (Awad & Khanna, 2015). Given training vectors $x_{i}\in R^{p},I=1,\cdots ,n$, and a vector $y\in R^{n}$, ε‐SVR solves *W* and *b* which are coefficients estimated following the optimization process:

$$\mathrm{Minimize}:\frac{1}{2}{\left\|W\right\|}^{2}+C\frac{1}{N}\sum_{i=1}^{N}\left(\xi_{i}+\xi_{i}^{*}\right)$$

where $y_{i}-wx_{i}-b\leq \varepsilon +\xi_{i}$, $wx_{i}+b-y_{i}\leq \varepsilon +\xi_{i}^{*}$ and $\xi_{i},{\;\xi }_{i}^{*}\geq 0.$

Similar to *λ* in regularization regression, *C* is a penalty parameter that gives weight to minimizing the flatness or error. The $\left(\xi_{i}+\xi_{i}^{*}\right)$ term represents the empirical loss term that considers only a subset of training points which are called support vectors for the model and *ε* is the threshold value that controls the number of support vectors used in model fitting (Awad & Khanna, 2015). An extremely small ε indicates a large number of support vectors and would ultimately lead to a less generalizable model regardless of good performance. Hence, the levels of training performance and model complexity highly depend on both *C* and *ε*.

Among multiple kernel functions including linear, polynomial, and Gaussian kernels, the Gaussian kernel is the one that can efficiently manage nonlinearity of data and was therefore selected for the prediction model. The Gaussian kernel is based on the following equation:

$$K\left(x,x^{\prime }\right)=\exp\left(-\frac{{\left\|x-x^{\prime }\right\|}^{2}}{2\sigma^{2}}\right)$$

#### 2.4.6 Random forest regression

The idea of the random forest algorithm is based on combining the forecasts from numerous decision trees into one single model. Combining across decision trees serves to reduce bias from the training set that could result from overfitting on a single decision tree. Instead, in making a prediction, the random forest regression model takes the average of all the individual decision tree estimates.

Random forest model is an ensemble method that generates a number of decision trees in parallel in order to balance between underfitting and overfitting to produce a generalizable model. Training a random forest model mainly involves two statistical techniques: bootstrapping and bagging. First, N bootstrapped sample sets are obtained from the entire training set by random sampling with replacement. Each set is then used to construct a regression tree without limiting the number of decision trees in order to enable the tree to reach its best performance of predicting the target. Instead of considering all features, only a smaller and fixed number (*m*) of total features would be considered as split candidates, as using only a subset of the predictors can eliminate the correlations among all N trees. The variable *m* is generally recommended as the square root of the total number of predictors. Subsequently, bagging essentially averages all *N* trees to reduce the overall model variance (Awad & Khanna, 2015):

f^RFNx=1N∑k=1NTkx

where *x* is the vectored input variable and $T_{k}\left(x\right)$ denotes a single regression tree constructed based on a subset of features and the bootstrapped samples. *N* is the number of trees, which is a key parameter and requires tuning for optimal model performance. The other tuning parameter, max depth, is often applied considering the complexity and nonlinearity of the regression model. For regression problems, the target, SSRT, contains real valued numbers, and random forest fits the regression model to SSRT using samples of features. The data is split into several points for each of features, and the sum of square error (SSE) at each split point is calculated for predicted values and observed values and the minimum SSE is selected for the node. Overall, it operates by constructing a multitude of decision trees at training time and outputting the class that is the mean SSRT of the individual trees.

#### 2.4.7 Gradient boosting regression

Gradient boosting algorithm is another ensemble method that enhances the single tree‐based model by sequentially generating a series of trees. The major difference compared to random forest is a boosting algorithm in which each tree is constructed depending on the performance of the previous trees and the main objective is to assign larger weights to the observations that were fitted poorly in the previous tree.

### 2.5 Generalizability: Training and assessment

#### 2.5.1 Train test sample split

In order to obtain an unbiased estimate of the predictive model performance of the algorithm, a train‐test split was applied for this study. Participants were randomly divided into independent training (80%) and lockbox (20%) subsets. The training subset was used solely for the learning procedure of the predictive model algorithm and the lockbox subset was used to estimate the performance of the trained predictive model algorithm. Thus, the lockbox sample was not made available in the learning stage and was separated from the training data and served to determine whether overfitting had occurred in the model construction.

#### 2.5.2 Cross‐validation

K‐fold cross‐validation was used to assess each model's generalizability. The training data were first divided into K subsets, one of which was used as a validation set, and models were trained on the remaining K‐1 subsets. Model errors were averaged over K iterations. In this study, fivefold cross‐validation was selected to randomly split the training data set.

In order to evaluate the accuracy of each model, the coefficient of determination, R‐squared score ($R^{2}$), was used to assess the deviation between the predicted and actual SSRT. $R^{2}$ represents the proportion of variance of actual SSRT values that a model's SSRT predictions explain and compares the fit of the model with that of a sample mean null hypothesis. If the chosen model has higher error on the validation set than the sample mean, then $R^{2}$ is negative. In ML studies, it is not uncommon for models to have negative validation $R^{2}$, which indicates that the model does not generalize.

Using a frequentist approach, we were able to run a paired‐test and compute the *p* value to determine whether a predictive model was significantly better than an alternative predictive model with different features or algorithms using the Diebold–Mariano test (Dietterich, 1998). This corrected paired *t* test is computed as (Bouckaert & Frank, 2004):

$$t=\frac{\frac{1}{k∙r}\sum_{i=1}^{k}\sum_{j=1}^{r}x_{\mathrm{ij}}}{\sqrt{\left(\frac{1}{k∙r}+\frac{n_{\text{test}}}{n_{\text{train}}}\right){\hat{\sigma }}^{2}}}$$

where *k* is the number of folds, *r* is the number of repetitions in the cross‐validation, *x* is the difference in performance of the models, $n_{\text{testing}}$ is the number of samples used for testing, and $n_{\text{train}}$ is the number of samples used for training.

#### 2.5.3 Hyperparameter optimization

In this study, random search, the most common technique for optimizing multiple parameters simultaneously, was implemented to tune and select the optimal parameters. The algorithm randomly selected 100 hyperparameter combinations in SVM, random forest, and gradient boosting regression through a discrete grid with combinations of reasonable values for each parameter and output the combination that maximizes $R^{2}$. In this search pattern, random combinations of parameters are considered in every iteration.

#### 2.5.4 Feature importance

The beta weights were computed for each feature of the best predictive model; a feature's beta weight represents its contribution to the model. However, for random forest and gradient boosting, entropy as a metric for calculating uncertainty and information gain measures how uncertainty in the target variable is reduced, given a set of independent variables.

In this study, whether neuroimaging data can estimate individual differences on performance in SST was the primary focus and, thus, covariates like age and sex were not added to the model even though they might be able to boost the prediction results. The rationale is that these variables are likely confounded with brain differences of interest. Due to their shared variance with the brain measures, it is likely that these variables might dominate (and overshadow) brain predictors which would affect our primary goal to quantify the maximum extent to which brain activation can explain SSRT. That said, analyses were run separately for males and females and returned comparable effect sizes (Table S8), neither of which were as large as when the sexes were combined in one analyses due, presumably, to the increased sample size of the combined analyses.

## 3 RESULTS

### 3.1 General ML results

As expected, most linear regression models were unable to provide good SSRT prediction on the validation sets, as indicated by negative validation $R^{2}$ values (see Table S2). The negative $R^{2}$, in the presence of training set *R* ^2^ values of 100%, indicates severe model overfitting in which the models fit to the idiosyncrasies of the training data and, consequently, have poor generalizability. Most of the ML algorithms returned $R^{2}$ scores greater than simple linear regression for each data set (Figure 1). The highest $R^{2}$ values on the validation sets among the ML methods was 10.92% with ridge regression and 10.74% with elastic net regression using data for all modalities and 10.07 and 10.08% for SST activation data alone with ridge and elastic net respectively (see Table S2). While ridge and elastic net regression were able to explain relatively high amounts of variance in SSRT, they did not significantly outperform other regression methods on the SST data sets (see Table S4). Similarly, random forest regression showed a relatively poor result compared with the other ML methods (see Table S4), but it is not significantly predicting less than other algorithms after Bonferroni correction.

### 3.2 Modalities comparison

**FIGURE 2:**
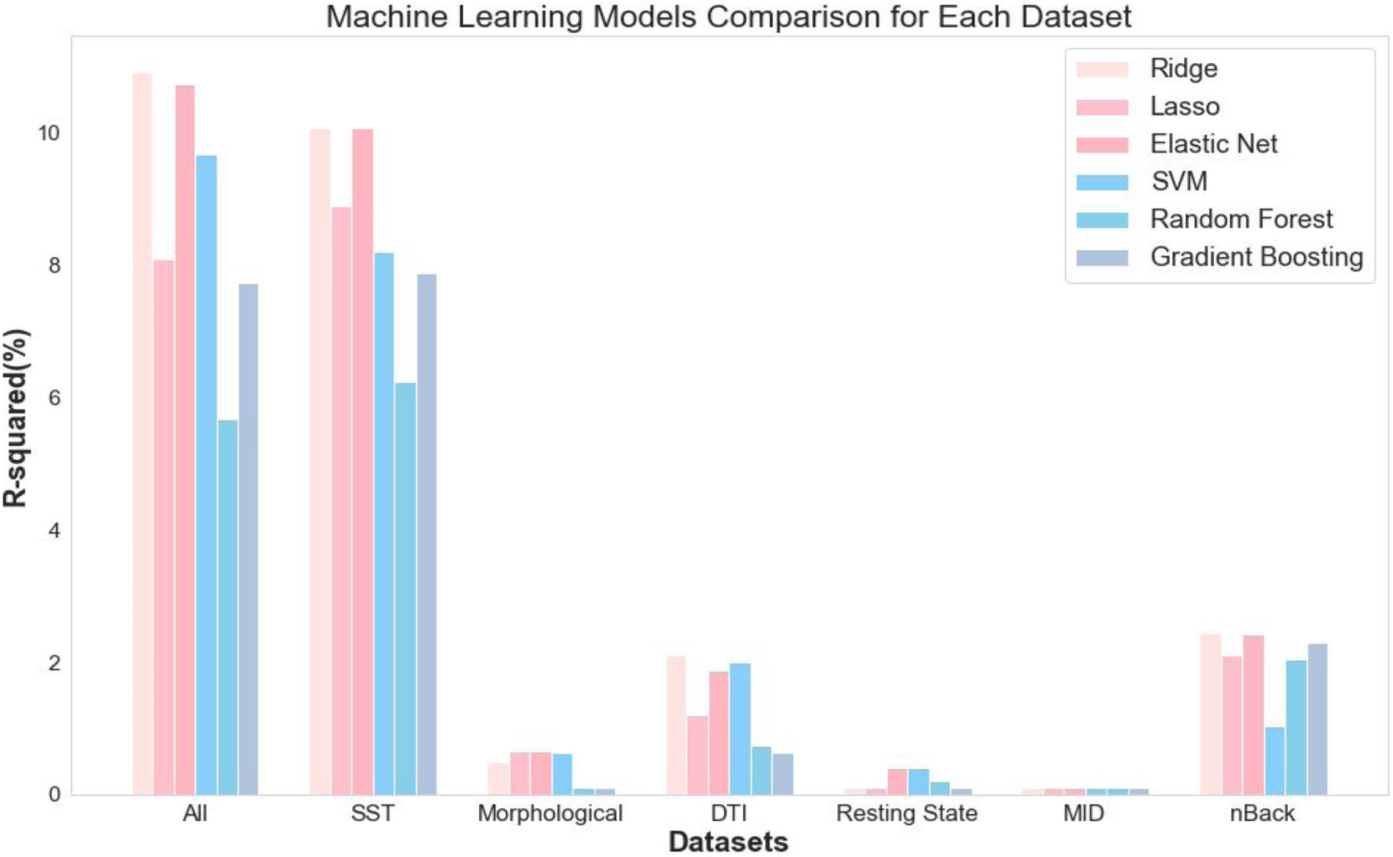
Proportion of variance in stop‐signal reaction‐time (SSRT) explained by each model averaged across the fivefold cross‐validation analyses. Results with *R* ^2^ score lower than zero are presented as zero in this plot

Notwithstanding the generally low effectiveness of linear regression, most algorithms performed better at predicting SSRT with the SST activation data compared to the other individual data sets (see Figure 2). Due to elastic net regression having the highest or second highest predictability among different regression models for each modality (see Table S3), it was selected as the algorithm to compare each pair of modalities with fivefold cross validation predictions. The high performance of the SST data set, even in comparison to the All‐modalities data set, confirmed expectations that the SST would dominate in estimating SSRT. Among the other task fMRI data sets, the EN‐Back performed better than the MID (*t* = 3.26, *p* = .02) while among the structural measures, the DTI data performed better than the morphological measures (*t* = 3.94, *p* < .01, see Table S4).

### 3.3 SST: Regional and contrast comparisons

With confirmation of SST activation data being the strongest predictor of SSRT, we focused next on an in‐depth exploration of this data set using all participants with SST data that passed quality control and all exclusion criteria (*n* = 7298). The increased sample size improved model performance for all algorithms except random forest (see Table 2) although the improvement for the best model (elastic net regression) was modest (*n* = 7298, *R* ^2^ = 11.92%; *n* = 3290, *R* ^2^ = 10.08%). Ridge regression and SVM regression both had notably strong performance with $R^{2}$ of 11.86 and 11.70%, respectively.

**TABLE 2:** Proportion of variance in SSRT explained by each model averaged across the fivefold cross‐validation analyses. The different data sets included all features as well as subsets of features from the cortical, subcortical, left, and right hemisphere parcellations

Regression model	All SST features	Cortical	Subcortical	Left hemisphere	Right hemisphere
Linear	4.47	4.57	2.06	7.87	5.97
Ridge	11.86	11.09	2.83	9.99	9.19
LASSO	11.41	10.27	3.06	10.19	9.40
Elastic net	11.92	11.06	3.00	10.08	9.33
SVM	11.70	10.72	3.84	10.30	9.65
Random forest	5.31	5.31	3.39	5.09	4.84
Gradient boosting	10.70	10.69	3.04	9.73	7.81

Abbreviations: SSRT, stop‐signal reaction‐time; SST, stop‐signal task.

To identify subsets of brain features particularly valuable for capturing individual differences in SSRT, we examined a number of meaningful feature groups (split by contrasts, cortical–subcortical, and left–right hemispheres) across the different ML algorithms. Since the elastic net model performed best on predicting SSRT (see Table S2) it was the chosen algorithm for statistical comparison among different feature groups. Across fivefold cross validation scores, prediction was superior (*t* = 23.53, *p* < .01) with cortical (max *R* ^2^ = 11.09%) compared to subcortical features (max *R* ^2^ = 3.84%). Similarly, performance restricted to left hemisphere features was slightly better than performance with right hemisphere features but the differences for the elastic net model were modest (*t* = −1.22, *p* = .14).

### 3.4 Contrasts comparison

**FIGURE 3:**
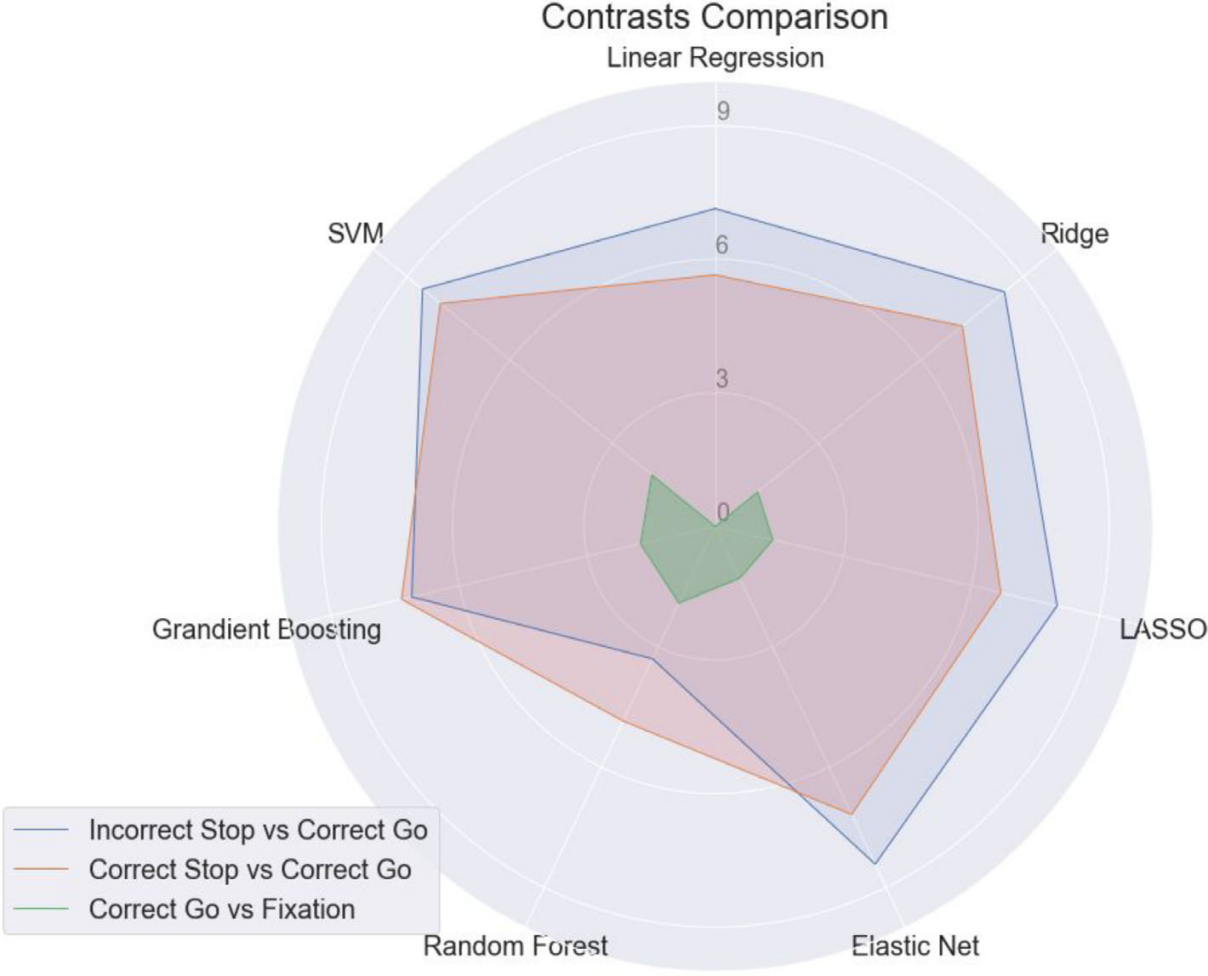
Response (incorrect stop vs. correct go) has stronger predictability on stop‐signal reaction‐time (SSRT) than success response (correct stop vs. correct go) and go trials response (correct go vs. fixation) with less variance predicted

When comparing different SST contrasts, stop‐related contrasts performed better than Go‐related contrasts (see Table S3). Elastic net regression was the best performing algorithm using all three contrasts (11.92%) and showed similar accuracies for stop fail and stop success contrasts (8.42 vs. 7.18%, respectively, *t* = 0.87, *p* = .22), both of which were significantly greater than the Go response contrast (1.29%; *t* = 6.28, *p* < .01 [stop success]; *t* = 13.51, *p* < .01 [stop fail]). This pattern held across all algorithms (see Figure 3).

### 3.5 Lockbox set approach and feature importance

**FIGURE 4:**
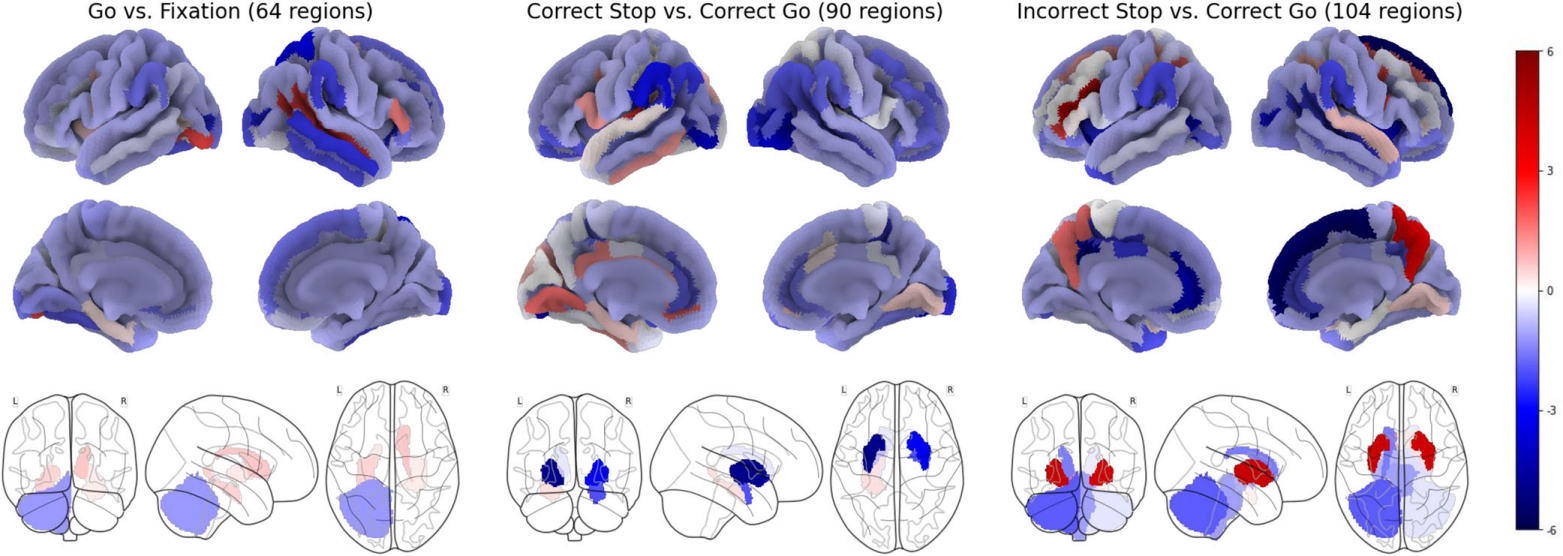
Positive and negative beta weights for features that survived in the final elastic net regression model

**FIGURE 5:**
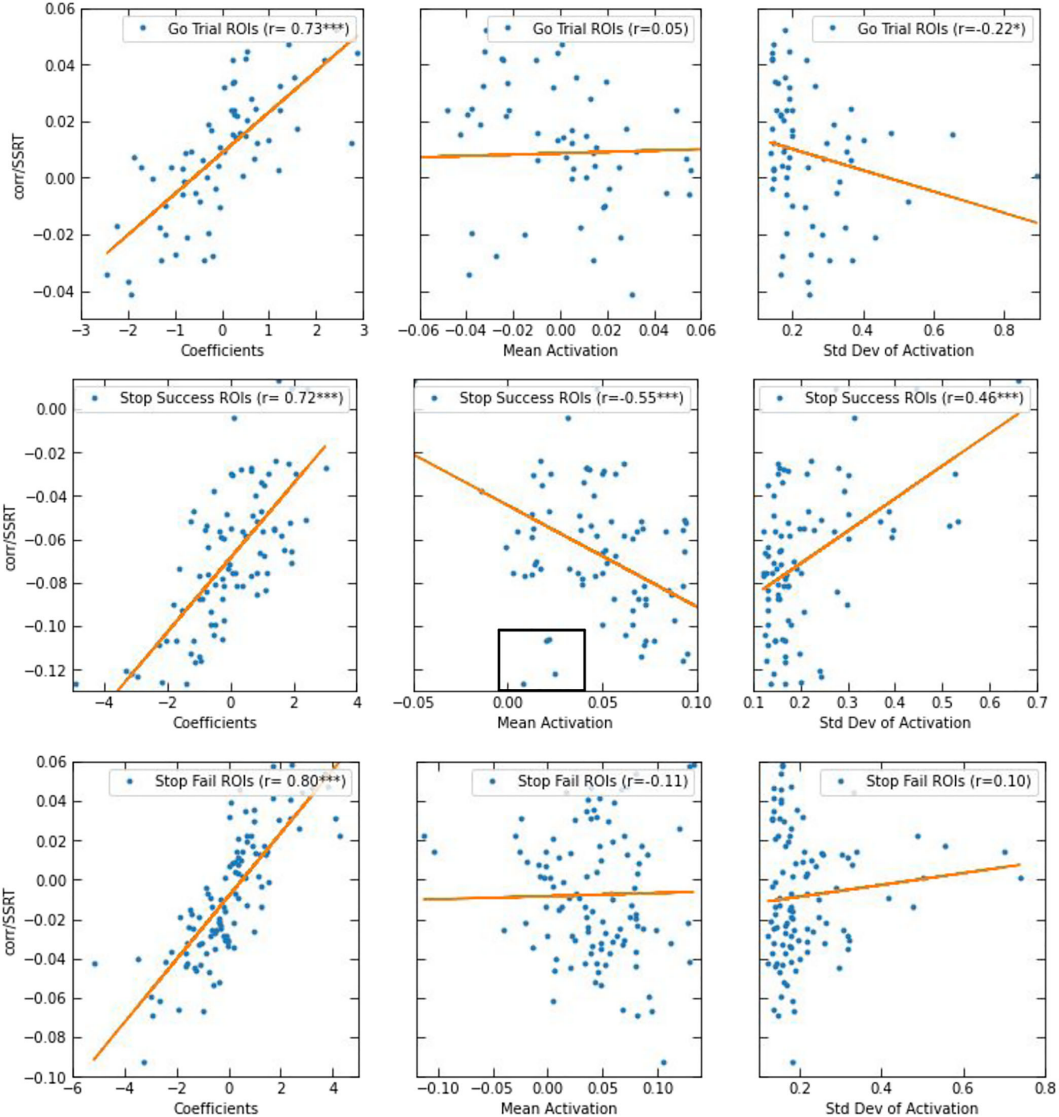
The scatterplots display the pairwise correlations with stop‐signal reaction‐time (SSRT) of the features in the final predictive model separated by the three task contrasts. Each feature's correlation with SSRT is plotted against their coefficients in the predictive model (left), mean activation (middle), and the standard deviation of activation (right). (***p < .01, **p < .05, *p < .1)

As the model that contained all three SST contrasts was most successful in explaining interindividual variance in SSRT (11.92% in the k‐fold cross‐validation using elastic net regression), we evaluated its performance on the set‐aside 20% lockbox set and examined the features that contributed to its performance (See Figure 4). Performance on the set‐aside 20% lockbox set was very similar to the cross‐validation performance (go trial contrast: 1.11%; stop success contrast: 7.79%; stop fail contrast: 7.68%; all three contrasts: 12.54%). As elastic net regression can eliminate features with small effects, the surviving features (258 from the initial set of 495 features) can all be viewed as contributing to the model's success. Figure 5 shows that go trials contributed the fewest (64) features, followed by stop success (90), and then stop fail (104). Among these features, regions such as bilateral putamen on successful stop trials and regions in the frontal lobe on both stop success and stop fail trials had negative coefficients in the model, indicating shorter SSRT (faster inhibition) when these regions were more active; in contrast, SSRT increased (slower inhibition) when regions like bilateral putamen were more active on stop fail trials.

In order to understand the characteristics of the final model's features (regions), we plotted the relationships between each feature's correlation with SSRT (corr/SSRT) and its coefficient in the final predictive model, its mean activation, and its standard deviation of activations (reflecting interindividual variation in activation) in Figure 5. For all three contrasts, the feature's coefficients in the model were highly correlated with the feature's pairwise correlations with SSRT (stop success: *r* = .72, *p* < .01; stop fail: *r* = .80, *p* < .01; go trial: *r* = .73, *p* < .01). This indicates that the magnitude of each feature's contribution in the predictive model is tightly coupled with how closely linked that feature, in isolation, is with individual differences in SSRT. Next, the correlation between corr/SSRT and the mean activation of each feature is significant for stop success (*r* = −.55, *p* < .01) but not for stop fail (*r* = −.11, *p* = .245) nor go trial (*r* = .05, *p* = .69). This result demonstrates that brain regions that are most activated by the task are also more related to individual differences in performance on the task, but this is only true for activations when successfully inhibiting. Finally, the standard deviations of activation levels of regions were positively correlated with corr/SSRT for stop success (*r* = .46, *p* < .01), uncorrelated for stop fail (*r* = .10, *p* = .30), and negatively, but not significantly, correlated for go trials (*r* = −.22, *p* = .08). This pattern suggests that regions with more interindividual variation when successfully inhibiting were better at explaining interindividual variation in SSRT. Conversely, the more interindividual variation a region showed on go trials, the worse it was in capturing interindividual variation in SSRT.

**FIGURE 6:**
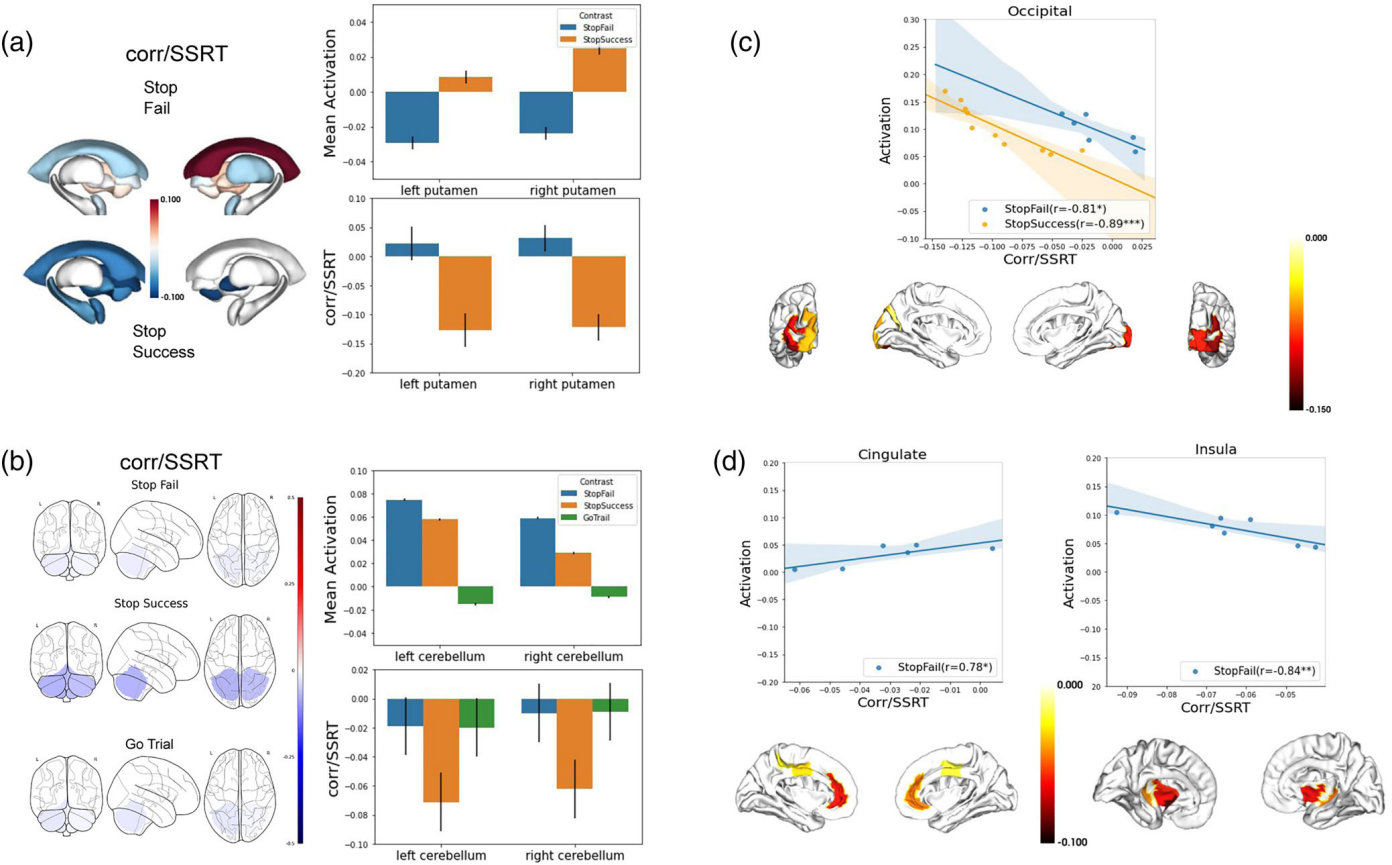
Analyses revealed involvement of widespread brain regional activations underlying individual differences in inhibitory control. (a) Subcortical regions that correlated with stop‐signal reaction‐time (SSRT) and bar graphs showing putamen activations and correlations with SSRT for both stop fail and stop success activations. (b) Bilateral cerebellum activation levels on the three task contrasts and their correlation with SSRT. (c) Regions in the occipital lobes were among the most activated areas and showed a very tight coupling between activation levels and their strength of correlation with SSRT. (d) Cingulate and insula activation levels and their correlation with individual differences in SSRT

There were a number of brain features with notable properties. As shown in Figure 5 (in set box) and Figure 6a, a set of subcortical areas including bilateral putamen, left caudate, and right amygdala were not strongly activated on stop success trials but nonetheless performed well in capturing individual differences in SSRT prediction. Moreover, as shown in Figure 6a, the putamen was positively activated on stop success trials, with higher activation, across participants, being related to faster SSRT; however, this same region was deactivated on stop fail trials with the level of deactivation also being related to faster SSRT. Cerebellar activation was high on stop success and stop fail trials but not on go trials (Figure 6b) and only stop success trials had a significant negative (*p* < .01) correlation with SSRT.

As noted in Figure 5, there was a significant relationship between a region's activation levels on stop success trials and its correlation with SSRT. This was especially true for regions in visual cortex. The scatterplot in Figure 6c shows this relationship for stop success activation in the seven occipital regions included in the final model and stop fail activation for the 10 occipital regions included in the final model. The notably high correlations here indicate that activations in these early visual processing areas were very tightly coupled to the contribution that region made to explaining individual differences in motor inhibition. Moreover, five visual regions were among the top ten highest regional correlations between activation and SSRT. Finally, stop fail activations were strongest in parietal and visual areas. However, insular and cingulate regions, key elements of the salience network, were also highly active. The insular regions showed a significant negative correlation (*r* = −.84, *p* = .016) between their activation levels and their correlation with SSRT indicating that the more active an insular region was on error trials, the better it performed in explaining individual differences in SSRT.

## 4 DISCUSSION

### 4.1 Model performance evaluation

In this study, we investigated the relationship between stop task performance and structural and functional neuroimaging measures. Exploring multiple ML algorithms enabled us to assess any advantages to be obtained by combining feature elimination and feature coefficient reduction and doing so in the current analysis resulted in a model that was able to predict 12.54% of the variance in SSRT in the set‐aside 20% lockbox.

Elastic net regressions yielded the most accurate predictions but both ridge regression and SVM both generated comparable estimations. There was no significant difference between SVM and the elastic net regression model, indicating that linear associations provided sufficient information for the predictive models. We also evaluated adding a first‐step univariate feature (ROI) selection approach (Table S9). Although the best model with feature selection (pairwise correlations of each brain variable against SSRT) was able to explain 11.22% of the variance in SSRT (this SVM model contained 300 features) this was not an improvement over the elastic net regression on all features (11.92%) due presumably to the feature selection that is an inherent part of the algorithm (the elastic net returned a model containing 258 features).

### 4.2 Evaluation of MRI modalities and subsets of brain features

Although some concerns regarding the SST implemented in the ABCD study have been suggested (Bissett et al., 2020), they have been shown to have minimal impact on the brain imaging data (Garavan et al., 2022). The present study further indicates that SST activation in the ABCD study is capable of capturing individual differences in response inhibition. As expected, we found that SST activation predicted SSRT better than any other brain measure examined. For the rest of the neuroimaging data types, all algorithms showed relatively poor ability to predict SSRT (although the DTI data showed modest accuracy). From an analytic perspective, a comparison of the all‐modalities and SST‐only results suggests that it is not always ideal to include as many features as possible in an ML framework; unnecessary features can introduce noise, and having too many features can encourage overfitting, producing a less generalizable model (see Table S2). If a smaller number of features provides similar accuracy to a model with a larger number of features, it is usually better to use the model with fewer features. Thus, despite the apparent superior performance (not statistically significant) of the all‐modalities elastic net model, we elected to focus on the SST fMRI measures.

Interrogating the SST data set, analyses indicated that cortical measures were more effective than subcortical measures in predicting inhibitory control, indicating that cortical data may be more sensitive to individual differences in this cognitive process. Indeed, numerous studies have found that activation during response inhibition is observed in multiple cortical regions including the IFG, pre‐SMA, and dACC (Nachev et al., 2007). It is also notable that the left and right hemispheres predicted SSRT equally well, with each hemisphere predicting around 10% of the variance in interindividual variation in SSRT. Previous studies have demonstrated that although both right and left hemispheres are capable of response inhibition (D'Alberto et al., 2017), there is evidence suggesting a superiority to the right hemisphere (Chambers et al., 2006; D'Alberto et al., 2017; Lenartowicz et al., 2011; Swann et al., 2009). As there is evidence that inhibitory processes are more right dominant in adults compared to children (Rubia et al., 2006), it will be interesting to determine if the relative importance of the two hemispheres for predicting SSRT scores changes as the sample ages and neurocognitive functions become more lateralized.

### 4.3 Brain regions underlying individual differences in SSRT

In general, coefficients of the predictive model were highly correlated with the pairwise correlations between brain activations and SSRT (see Figure 6). Although there were exceptions such as the bilateral putamen, the general pattern was that the brain regions that were most correlated with SSRT tended to be those showing both more stopping‐related activation and more variation across participants. As the most strongly correlated region with SSRT explained just 2% of the variance (anterior occipital sulcus with *r* = −.13), the present results underscore the importance of multivariate analysis for capturing individual differences.

Although subcortical fMRI measures of the SST in isolation explained less than 4% of the variance in SSRT (Table S2), bilateral putamen activation on both stop success and stop fail trials were among the most important features predicting SSRT (i.e., had the highest beta weights; Figure 4) in models incorporating both subcortical and cortical measures. Although activation levels in the putamen, in addition to other subcortical regions such as the left caudate and right amygdala, had notably high correlations with SSRT they were not among the highly active brain regions (at the group average level). As the striatum contributes to both the direct and indirect cortical–subcortical pathways that contribute to motor inhibition (Jahfari et al., 2010; Jahfari et al., 2011; Lanciego et al., 2012; Nambu et al., 2002), this result likely reflects individual differences in reliance upon these various pathways. Cerebellar activation on stop success trials was also related to SSRT although activation levels were actually higher on stop fail than stop success trials. Perhaps consistent with this observation, a recent investigation into the cerebellum's role in inhibitory control concluded for an important role in detecting errors in movement by comparing the predicted with the actual sensorimotor consequences of a movement and may regulate inhibitory control by forecasting errors or actions related to errors (Mannarelli et al., 2020).

Visual regions were prominent among stop success activations for having both relatively high levels of activation as well as being relatively highly correlated with SSRT. The results suggest that the visual processing of the task stimuli, including the possibility of their top‐down attentional modulation, may contribute in important ways to task performance at this age. Across the cortical visual regions in the final model, the association between activation levels and the magnitude of the correlation between the region's activation and SSRT was *r* = −.89 for stop success and *r* = −.81 for stop fails (see Table S5). Although the correlations between activation in each ROI and SSRT were relatively modest (*r* = −.14 to .02), this result indicates that the magnitude of activation in these visual regions contributes very substantially to how closely coupled that region is to individual differences in motor inhibition.

A number of cingulate regions were activated on stop fail trials and showed negative correlations between activation levels and SSRT. A large body of research links the dACC to cognitive control, especially regarding the monitoring of suboptimal performance (Carroll et al., 2015; Le et al., 2021; Luijten et al., 2014) but also impulse control (Borst et al., 2014; Klein et al., 2013). Similarly, the insula, implicated in the interoceptive and subjective aspects of error detection (Cai et al., 2014; Ham et al., 2013) was activated on stop fail trials and activation here was also correlated negatively with SSRT. Indeed, eight of the top 10 strongest negative correlations with SSRT were in cingulate and insular regions, suggesting that the neural processing of errors is related to better inhibitory control abilities through, for instance, post‐error motor slowing (Cavanagh et al., 2010).

For go trials, the positive, albeit weak, association that was observed between activation levels and the correlation with SSRT suggested that longer SSRTs were linked to greater activation during go responses. In contrast, the two regions with the highest anti‐correlation between Go activation and SSRT were also within occipital cortex, echoing the finding above that the visual processing of the Go stimulus is related to being faster to inhibit. Indeed, the effects of attention on behavioral performance (e.g., shortened RT, increased accuracy) are related to increased occipital cortex activity (Kanwisher & Wojciulik, 2000; Luck, 2014; Prime & Jolicœur, 2009; Ungerleider, 2000). However, the current study focuses on response inhibition, although a more complete assessment of cognitive control on this task might incorporate other processes such as attentional orienting (Chao et al., 2009; Duann et al., 2009), error detection (Li et al., 2008) and post‐error slowing (Chang et al., 2017; Ide & Chiang‐Shan, 2011; Zhang et al., 2017). These processes were not incorporated in the present analyses due to a lack of targeted contrasts for them in the ABCD SST brain activation data, but they could be computed, and would represent a likely fruitful extension of the present work.

## 5 CONCLUSION

This study has demonstrated the efficacy of a multivariate approach for capturing individual differences in inhibitory abilities. While a notable improvement over previous univariate results, the success of the elastic net algorithm in explaining approximately 12% of variance in out‐of‐sample participants clearly indicates that there is substantial room for improvement. While improved prediction may result from better measures including, for example, more reliable estimates of task activation or inhibitory abilities, other analytic strategies such as stratification of participants (based on demographic, psychological or neurobiological factors) or incorporation of other relevant features such as inter‐regional task connectivity offer potential. Importantly, determining how intraindividual and interindividual differences develop throughout adolescence as the ABCD sample ages will be key to understanding how this neurocognitive process influences, and is influenced by, a multitude of psychosocial factors. There are several limitations to this study that should be highlighted. First, the study was restricted by the limited number of SST contrasts that probe only the main aspects of the task (go and stop success and failure), although current studies suggest that other cognitive processes such as proactive control of motor responses are relevant to SSRT (Hu et al., 2015; Hu et al., 2016; Hu & Li, 2012; Wang et al., 2018). To achieve a better predictive model on individual performance in SSRT, such processes should be included in the ML model. Also, only ROI‐level data were examined rather than voxelwise data in this study as ROI data are easily interpretable and provide a relatively small feature set size. Beyond task activation, task functional connectivity measures (Cole et al., 2013; Finn et al., 2017; Greene et al., 2018; Jiang et al., 2020; Mennes et al., 2010) also have great potential for estimating individual differences in future prediction analysis. Furthermore, the current results from 9‐ and 10‐year‐olds participants in the ABCD study may not represent adolescents at other ages. As a longitudinal study, ABCD Study offers an important opportunity to explore individual differences in response inhibition in older children. Regions of prefrontal cortex may be critical features in the predictive model with children in their late adolescent period.

## Supporting information


**TABLE S1** Violators are participants whose mean failed stop response reaction time is greater than their mean go reaction time. Glitch subjects are those who suffered from an SSD programming glitch. Subjects with many 0 SSD are participants with over 20 zero‐SSD trials. Performance flagged is participants who did not have an acceptable performance in the task. Outliers are participants with a measure which is 10 standard deviations or more away from the mean value. Total exclusion (overlapped) is the final number of participants excluded for any of the exclusion‐types listed in the table.
**TABLE S2** Proportion of variance in SSRT explained by each model averaged across the fivefold cross‐validation analyses.
**TABLE S3** *R* ^2^ (%) of predicting SSRT for each model on SST data set as well as three different contrasts
**TABLE S4** T‐statistics between each pair of modalities across 5 folds R‐square performance (***p < .01, **p < .05, *p < .1)
**TABLE S5** T‐statistics between each pair of algorithm across 5 folds R‐square performance predicting SSRT with SST (***p < .01, **p < .05, *p < .1)
**TABLE S6** Characteristics of Stop success occipital regions
**TABLE S7** Top 10 negative corr/SSRT with stop fail
**TABLE S8** *R* ^2^ (%) when predicting SSRT separately for males and females. One‐tailed *t*‐test comparing male and female model results across the six algorithms was not statistically significant *t*(5) = 1.514, *p* = .08.
**TABLE S9** *R* ^2^ (%) for predicting SSRT across numerous algorithms and with 10 different univariate feature selection groups.

## Data Availability

Data used in the preparation of this article were obtained from the ABCD Study (https://abcdstudy.org) held in the NDA. This is a multisite, longitudinal study designed to recruit more than 10,000 children ages 9–10 years old and follow them over 10 years into early adulthood. The ABCD study is supported by the National Institutes of Health and additional federal partners under award numbers U01DA041048, U01DA050989, U01DA051016, U01DA041022, U01DA051018, U01DA051037, U01DA050987, U01DA041174, U01DA041106, U01DA041117, U01DA041028, U01DA041134, U01DA050988, U01DA051039, U01DA041156, U01DA041025, U01DA041120, U01DA051038, U01DA041148, U01DA041093, U01DA041089, U24DA041123 and U24DA041147. A full list of supporters is available at https://abcdstudy.org/federal-partners.html. A listing of participating sites and a complete listing of the study investigators can be found at https://abcdstudy.org/consortium_members/.
