# Supplementary material for: Machine learning approaches linking brain function to behavior in the ABCD STOP task
Source: Hum Brain Mapp. 2022 Dec 19;44(4):1751–66. doi: 10.1002/hbm.26172 (PMC9921227; doi:10.1002/hbm.26172)
Supplement: Supplementary file 1 — TABLE S1 Violators are participants whose mean failed stop response reaction time is greater than their mean go reaction time. Glitch subjects are those who suffered from an SSD programming glitch. Subjects with many 0 SSD are participants with over 20 zero‐SSD trials. Performance flagged is participants who did not have an acceptable performance in the task. Outliers are participants with a measure which is 10 standard deviations or more away from the mean value. Total exclusion (overlapped) is the final number of participants excluded for any of the exclusion‐types listed in the table. TABLE S2 Proportion of variance in SSRT explained by each model averaged across the fivefold cross‐validation analyses. TABLE S3 R 2 (%) of predicting SSRT for each model on SST data set as well as three different contrasts TABLE S4 T‐statistics between each pair of modalities across 5 folds R‐square performance (***p < .01, **p < .05, *p < .1) TABLE S5 T‐statistics between each pair of algorithm across 5 folds R‐square performance predicting SSRT with SST (***p < .01, **p < .05, *p < .1) TABLE S6 Characteristics of Stop success occipital regions TABLE S7 Top 10 negative corr/SSRT with stop fail TABLE S8 R 2 (%) when predicting SSRT separately for males and females. One‐tailed t‐test comparing male and female model results across the six algorithms was not statistically significant t(5) = 1.514, p = .08. TABLE S9 R 2 (%) for predicting SSRT across numerous algorithms and with 10 different univariate feature selection groups. [file HBM-44-1751-s001.pdf]

Supplementary Material

Exclusion Type	Subjects Excluded
Violators	693
Glitch Subjects	258
Subjects with many 0 SSD	821
Performance flagged	1009
Outliers	422
Total exclusion (overlapped)	2244

Table S1 - Violators are participants whose mean failed stop response reaction time is greater than their mean go reaction time. Glitch subjects are those who suffered from an SSD programming glitch. Subjects with many 0 SSD are participants with over 20 zero-SSD trials. Performance flagged are participants who did not have an acceptable performance in the task.Outliers are participants with a measure which is 10 standard deviations or more away from the mean value. Total exclusion (overlapped) is the final number of participants excluded for any of the exclusion-types listed in the table.

R^2 (%)	All modalities	SST	Morphological	DTI	Resting-State	MID	Nback
**Linear**	-779.57	-13.67	-14.62	-9.30	-22.22	-42.16	-14.62
**Ridge**	10.92	10.07	0.47	2.10	-0.49	-0.86	2.44
**LASSO**	8.10	8.89	0.65	1.20	-0.20	-0.32	2.11
**Elastic Net**	10.74	10.08	0.64	1.88	0.39	-0.49	2.42
**SVM**	9.68	8.19	0.62	2.00	0.40	-0.40	1.02
**Random Forest**	5.66	6.23	-0.04	0.74	0.20	-0.87	2.03
**Gradient Boosting**	7.73	7.88	-0.36	0.63	-0.48	-1.13	2.29

Table S2 Proportion of variance in SSRT explained by each model averaged across the 5-fold cross-validation analyses.

R^2 (%)	All 3 Contrasts	Incorrect Stop vs. Correct Go (Stop Fail)	Correct Stop vs. Correct Go (Stop Success)	Correct Go vs. Fixation (Go Trial)
**Linear**	4.47	7.14	5.65	-2.04
**Ridge**	11.86	8.45	7.22	1.24
**Lasso**	11.41	8.01	6.69	1.35
**Elastic Net**	11.92	8.42	7.18	1.29
**SVM**	11.70	8.55	8.03	1.85
**Random Forest**	5.31	3.30	4.85	1.91
**Gradient Boosting**	10.70	7.11	7.35	1.75

Table S3 R^2(%) of predicting SSRT for each model on SST dataset as well as three different contrasts

T-stats	All modalities	SST	Morphological	DTI	Resting State	MID
**SST**	-1.83*	-	-	-	-	-
**Morphological**	8.35***	8.47***	-	-	-	-
**DTI**	6.74***	6.86***	-3.94***	-	-	-
**Resting State**	8.09***	9.63***	1.41	6.71***	-	-
**MID**	12.49***	12.55***	3.54**	14.50***	2.29**	-
**Nback**	10.79***	10.96***	1.64*	7.71***	0.18	-3.26**

Table S4 T-statistics between each pair of modalities across 5 folds R-square performance (***p<0.01,**p<0.05, *p<0.1)

T-stats	Ridge	Lasso	Elastic Net	SVM	Random Forest
**Lasso**	1.55*	-	-	-	-
**Elastic Net**	-2.00*	-5.24***	-	-	-
**SVM**	-0.82	-1.71*	0.49	-	-
**Random Forest**	2.07*	1.95*	7.90***	5.41***	-
**GBM**	1.15	1.05	1.24	1.18	0.909

Table S5 T-statistics between each pair of algorithm across 5 folds R-square performance predicting SSRT with SST (***p<0.01,**p<0.05, *p<0.1)

Mean Activation	corr/SSRT	Coefficients	StD of Activation	Features	Contrast
0.170111	-0.139319	-2.905542	0.167716	s.occipital.ant.rh	StopSuccess
0.153844	-0.126303	-1.403336	0.165594	s.occipital.ant.lh	StopSuccess
0.137757	-0.122921	-2.959946	0.241769	g.and.s.occipital.inf.rh	StopSuccess
0.129658	-0.120804	-3.316264	0.240038	g.and.s.occipital.inf.rh	StopSuccess
0.103119	-0.116867	-1.119837	0.165804	g.occipital.middle.rh	StopSuccess
0.088482	-0.097643	-0.232920	0.174225	g.occipital.middle.lh	StopSuccess
0.072749	-0.090388	-1.830900	0.298586	pole.occipital.rh	StopSuccess

Table S6 Characteristics of Stop success occipital regions

Mean Activation	corr/SSRT	Coefficients	StD of Activation	Features	Contrast
0.105092	-0.092726	-3.285159	0.181004	s.circular.insula.ant.lh	StopFail
0.081514	-0.068581	-2.921298	0.146265	g.insular.short.rh	StopFail
0.094580	-0.066560	-0.977663	0.184859	s.circular.insula.ant.rh	StopFail
0.068708	-0.065679	-1.937457	0.136006	g.insular.short.lh	StopFail
0.004794	-0.061883	-2.648221	0.181722	g.and.s.cingul.ant.lh	StopFail
0.092231	-0.058984	-2.969247	0.155526	s.circular.insula.sup.lh	StopFail
0.048451	-0.053143	-0.615594	0.167579	g.ins.lg.and.s.cent.ins.rh	StopFail
0.043412	-0.051748	-0.345663	0.161451	ventraldc.rh	StopFail
0.046277	-0.046999	-0.780316	0.149024	s.circular.insula.inf.rh	StopFail
0.006414	-0.045748	-1.270461	0.188979	g.and.s.cingul.ant.rh	StopFail

Table S7 Top 10 negative corr/SSRT with stop fail

R^2 (%)	Male	Female
**Ridge**	6.83%	4.57%
**Lasso**	2.76%	2.94%
**Elastic Net**	6.07%	4.57%
**SVM**	9.80%	5.73%
**Random Forest**	5.47%	4.87%
**Gradient Boosting**	3.72%	0.52%

Table S8 R^2(%) when predicting SSRT separately for males and females. One-tailed t-test comparing male and female model results across the six algorithms was not statistically significant t(5)=1.514, p=0.08.

R^2 (%)	Top10	Top20	Top50	Top100	Top150	Top200	Top300	Top350	Top400	All
**Ridge**	2.55%	2.34%	2.86%	3.68%	4.78%	5.83%	8.43%	8.57%	8.60%	10.07%
**Lasso**	2.47%	2.26%	2.96%	3.17%	4.50%	5.67%	8.14%	8.16%	8.39%	8.89%
**Elastic Net**	2.54%	2.34%	2.84%	3.60%	4.76%	5.95%	8.29%	8.43%	8.65%	11.92%
**SVM**	3.59%	4.99%	6.30%	7.90%	9.11%	9.91%	11.22%	10.81%	9.97%	8.19%
**Random Forest**	4.81%	5.13%	5.49%	5.89%	7.15%	7.29%	7.00%	7.01%	6.94%	6.23%
**Gradient Boosting**	-5.52%	-6.75%	6.21%	5.26%	8.49%	9.14%	9.11%	9.28%	9.89%	7.88%

Table S9 R^2(%) for predicting SSRT across numerous algorithms and with 10 different univariate feature selection groups.
